# Phytochemical Characterization and Assessment of Crude Extracts from *Justicia adhatoda* for Phytotoxic and Cytotoxic Activity

**DOI:** 10.1155/2024/1374346

**Published:** 2024-09-25

**Authors:** Muhammad Nasir, Roha Ramash, Hira Fatima, Sana Ashraf, Iqra Munir, Sundas Asghar, Muhammad Adnan, Atifa Masood, Sunbal Khalil Chaudhari

**Affiliations:** ^1^ Department of Botany The University of Lahore, Sargodha Campus, Sargodha, Pakistan; ^2^ Department of Zoology The University of Lahore, Sargodha Campus, Sargodha, Pakistan

## Abstract

**Background:**

The aim of the study was to investigate the cytotoxicity, phytotoxicity, and proximate and phytochemical analysis of methanolic extracts of the leaves of *Justicia adhatoda*.

**Methods:**

Methanolic leaf extract of *J. adhatoda* was screened for phytotoxic activity by using root length inhibition and seed germination assays. Cytotoxic activity was calculated using brine shrimp lethality bioassay. Plant extracts were also investigated for their proximate composition. The presence of several phytochemicals was tested by employing different methods.

**Results:**

Decrease in seed germination and root length, 62.67% and 83.11%, was proportional to the increasing concentration of the methanolic extract of the plant. Cytotoxicity assay results indicated that the methanolic extract possessed significant cytotoxic potential with an LC-50 of 217 *µ*g/ml. Proximate analysis revealed that the leaves of *J. adhatoda* contain 9.4% moisture, 90.6% dry matter, 19.25% crude protein, 4.5% crude fat, 8.0% crude fiber, and 11.5% total ash.

**Conclusion:**

Methanolic extracts of *J. adhatoda* leaves showed significant cytotoxic effects and may have potential use in medicine. The *J. adhatoda* foliar extract shows good inhibitory effects against seed germination and root growth. Therefore, it might be used as soil additive in crops to control weeds. Further research is required to detect and isolate phytotoxins from the plant that might replace synthetic herbicides with eco-friendly herbicides.

## 1. Background

Allelopathy is a common biological phenomenon in which one plant produces biochemicals that promote or inhibit the growth, survival, or development of other plants [1, 2]. Countries across the globe are facing difficulties in improving crop yield due to the competition between weeds and crops for nutrients and other resources. Weeds can reduce crop output by an average of 34%. The output of some essential crops has been reduced by weeds as follows: cotton 36%, maize 40%, potatoes 30%, rice 37%, and wheat 23% [3]. Synthetic herbicides often have adverse impacts on both the environment and humans. Phytotoxicity or allelopathy can be a safe alternative for long-term weed control [4]. That is why, the allelopathic and phytotoxic behavior of plants is gaining the attention of researchers as a substitute for chemical weed killers.

It is estimated that from 1981 to 2002, 60% of new chemicals used in cancer therapy were derived from natural plant products or their derivatives [5]. Consequently, screening conventional medicinal plants is essential for identifying and isolating novel cytotoxic chemicals for the treatment of different diseases affecting humans. The brine shrimp lethality (BSL) bioassay is a quick, simple, and economical method for cytotoxicity screening [6]. Gilani et al. inspected 81 medicinal plants in Pakistan and found that these plants have allelopathic potentials [7]. Michael et al. developed the BSL bioassay for cytotoxic analysis, later an association was found between brine shrimp toxicity and 9 KB (human nasopharyngeal carcinoma) cytotoxicity [8, 9].

The Acanthaceae family comprises 2500 species of shrubs, herbs and vines. These species have been categorized into 200 genera [10]. The Acanthaceae family is horticulturally significant and is grown for its magnificent flowers [11, 12]. The medicinal characteristics of the family have been thoroughly studied in traditional, folk, and modern medicine [13].

The genus *Justicia* is diverse and the largest genus in the Acanthaceae family, having more than 700 species. [14]. *Justicia* species are used to treat respiratory, gastrointestinal, and inflammatory disorders, viral fever, malaria, rheumatism, epilepsy, headache, diabetes, cancer, arthritis, mental disorders, and HIV [15]. *Justicia adhatoda* is known as Vasaka (Sanskrit) [16], Malabarnut (English), Adusa (Hindi) [17], Arusa (Urdu) [18], and Bhekkar (Punjabi) [19]. It is a shrub that is prevalent in Southeast Asia's tropical climates [20]. In Pakistan, it is distributed in KPK Province, Chitral, Hazara, Malakand, Swat, Punjab, Rawalpindi, Sind, and Karachi [21]. It is a perennial, evergreen, densely branching shrub (1.0 m to 2.5 m tall) with a bitter taste and pungent odor. The plant has white, pink, or purple flowers that present on opposite ascending branches [22]. It is an Ayurvedic medicinal herb used to cure colds, coughs, asthma, and TB [23]. Additionally, it possesses antispasmodic (bronchodilator) and expectorant effects. [24].

### 1.1. Taxonomic Status

Kingdom: Plantae, Class: Magnoliopsida, Order: Lamiales, Family: Acanthaceae, Genus: *Justicia*, Species: *adhatoda* [25].

In Unani and Ayurvedic medicine, *J. adhatoda* is a well-known plant medicine [26]. It is an Ayurvedic herb used to treat coughs, asthma, bronchitis, and common colds [24]. It is the source of medication “Vasaka” which is widely recognized in traditional medicine for its curative properties, especially in the treatment of bronchitis [27]. Antispasmodic, anti-inflammatory, bronchodilator, antibleeding, disinfectant, antijaundice, antidiabetic, fever reducer, and oxytocic are some of the worth mentioning medical properties of this plant [28]. This plant has astringent, diuretic, purgative, antiperiodic, and expectorant effects and is capable of liquefying sputum [29]. Its leaves, flowers, and roots are applied in herbal medicines to treat, cancer [30] and tuberculosis [31], and exhibit antihelmintic effects [32]. The leaf extract is reported to be useful in treating dysentery, diarrhea, and glandular tumours [32].

*J. adhatoda* has been extensively researched for its phytochemical and pharmacological properties. It is included in the class of herbal medicines that have a very strong traditional basis. *J. adhatoda* contains significant amounts of vasicine, vasicolone, vasicinone, and other alkaloids. The plants has antibacterial, antifungal, antitussive, anti-inflammatory, hepatoprotective, antiulcer, antiviral, abortifacient, thrombolytic, antimutagenic, hypoglycemic, and antioxidant properties [33]. After performing phytochemical evaluations on various parts of *J. adhatoda*, namely on bark, fruit, flower, root, wood, and even the whole plant, approximately 233 phytochemicals have been identified so far. Among them are 12 flavonoids, 33 alkaloids, 47 essential oils, 47 organic acids, 23 terpenes and steroids, 14 phenolic compounds, hydrocarbons including alkanes, alkenes, alkylamine, acetylene, naphthalene, naphthoquinone, fatty alcohol, and 59 miscellaneous chemicals [34]. Additionally, some macro and micro minerals have been found in plant, such as calcium (Ca), chromium (Cr), copper (Cu), iron (Fe), potassium (K), manganese (Mn), vanadium (V), and zinc (Zn). [35].

## 2. Materials and Methods

### 2.1. Sample Preparation and Experimental Details

Plant leaves were collected in September 2021 from the Soon Valley (32°58′N 72°15′E) and Chinji National Park (33°0′36.87″N 72°29′30.98″E), Punjab, Pakistan. The leaves were cleaned, dried (at room temperature), ground, and converted into a fine powder. In order to obtain a pure extract of the plant, 50 g of leaf powder was mixed with 500 ml of methanol. A pure extract of the plant was achieved after filtration and evaporation. The extract was weighed after being concentrated, labeled, and kept in small sterilized bottles at 4°C for further analysis.

### 2.2. Phytotoxic Effect

The phytotoxic activity of *J. adhatoda* was analyzed with the help of root length inhibition and percentage inhibition in seed germination assays [36, 37]. Five different concentrations (0, 10, 100, 500, and 1000 ppm) of methanol extract were used in this experiment. To execute these assays, sterilized seeds were placed in petri dishes lined with two filter papers, and 5 ml of each solution was added to each petri dish.

### 2.3. Determination of Root Length Inhibition

The filter paper was placed in each petri dish, and then 5 ml of five different concentrations of 70% methanol extract (0, 10, 100, 500, 1000 ppm) were poured into each dish with the help of a pipette. After evaporation of solvent, 5 ml of distilled water was added to these dishes. Ten seeds were put in each petri dish. Petri dishes were properly sealed and kept at 23°C for incubation. The measurement of the length of the roots of all these seeds was noted after one, three, and five days. The percentage of growth inhibition was measured by using the following formula:

(1) $$\begin{array}{c} \text{Growth inhibtion percentage}=\frac{\left(100\left[\text{PC}-\text{PT}\right]\right)}{\text{PC}}. \end{array}$$

### 2.4. Determination of Germination Percentage

One hundred seeds of radish and 70% methanol extract with five different concentrations (0, 10, 100, 500, and 1000 ppm) were used in this experiment. The germination rate of seeds was noted for five days. The percentage of germination was calculated by dividing the number of germinated seeds by the total number of seeds sown in petri dishes and multiplying by 100, as described by Bhardwaj [38]. Experiment was performed three times to calculate the mean values and standard deviation for all treatments.

### 2.5. Cytotoxic Effect

#### 2.5.1. Brine Shrimp Lethality Assay

Cytotoxic potential was evaluated by the brine shrimp lethality test following the protocol of Meyer et al. [9, 39]. Brine shrimp eggs were hatched into a small partitioned tank containing artificial sea water (38 g/L, pH = 8.5). The 70% methanol extract was used in three different concentrations (1000, 100, and 10 ppm) and taken into tiny sterile vials in triplicate. A Pasteur's pipette was used to add ten shrimp to each vial. The vials were kept under artificial light at room temperature, and surviving brine shrimp were counted after 24 hours. The resulting data were assessed by using the following equation:

(2) $$\begin{array}{c} \text{Mortality}\%=\left(\frac{\text{Pc}-\text{Pt}}{\text{Pc}}\right)100. \end{array}$$

Data were analyzed by probit analysis to evaluate the Lethal Dose 50 (LC50) at 95% confidence intervals.

### 2.6. Proximate Composition Analysis

Proximate analysis was carried out to determine the percentage of moisture content, protein, dry matter, ash content, fat, and crude fiber. Moisture content was quantified using Nancy Trautmann's method. The well-known Kjeldahl method was used to determine the crude protein percentage also described by Onwuka [40]. Fat extraction was performed using a Soxhlet apparatus. Acid-base treatments were used to estimate the crude fiber percentage. Dry matter was calculated using a protocol outlined in AOAC [41].

## 3. Qualitative Phytochemical Analysis of Plant Extracts

Qualitative phytochemical tests for alkaloids, flavonoids, coumarins, phenols, saponins, and tannins were performed according to the protocol of Ismail et al. [42].

## 4. Results and Discussion

Effect of methanolic extracts of *Justicia adhatoda* leaves on radish seed germination and root length.

### 4.1. Seed Germination Percentage

**Figure 1:**
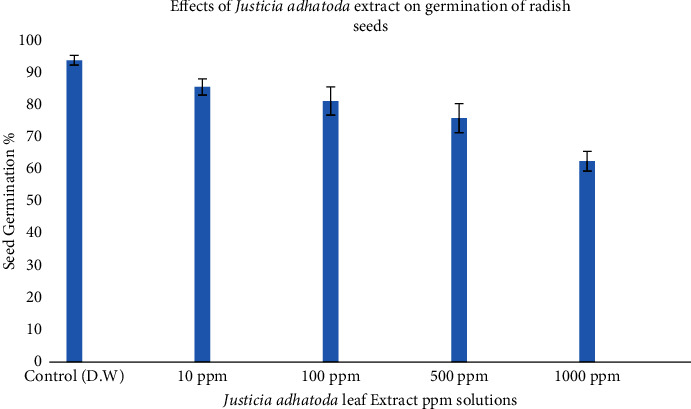
Effects of *Justicia adhatoda* extract on germination of radish seeds.

Results illustrate the allelopathic/phytotoxic effects of *J. adhatoda* extract on radish seed germination. Seed germination decreased by increasing the concentration of extract. Germination percentage decreases up to 62.33. Means of seed germination percentage are 93.67%, 85.33%, 81.00%, 75.67%, and 62.67% in 0 ppm, 10 ppm, 100 ppm, 500 ppm, and 1000 ppm extract, respectively (Figure 1 and Table 1).

Table 2 reveals the ANOVA on the effect of *J. adhatoda* extract on germination of radish seeds. A substantial difference was observed between the treated and control sample.

Similarly, Khan et al. assessed the phytotoxic activity of *J. adhatoda* against duckweed (*Lemna minor* L). They tested different fractions like crude methanolic extract, *n-*hexane, CHCl_3,_ ethyl acetate, and aqueous fraction [43]. These fractions proved to be phytotoxic and are consistent with our findings.

In a same way, Devkota et al. [44–46] studied that *J. adhatoda* and *Costus speciosus* had the highest inhibitory effects on seedling growth and germination of wheat, pea, and turnip, respectively. Furthermore, they observed that *J. adhatoda* plant extract potentially impaired anabolic activities in plants along with the visible inhibition on seed germination parameters [46, 47]. Our results are in harmony with previous studies conducted on phytotoxic effect of *J. adhatoda.* We have observed significant inhibitory effects on seedling growth in radish seed.

### 4.2. Root Length Inhibition

**Figure 2:**
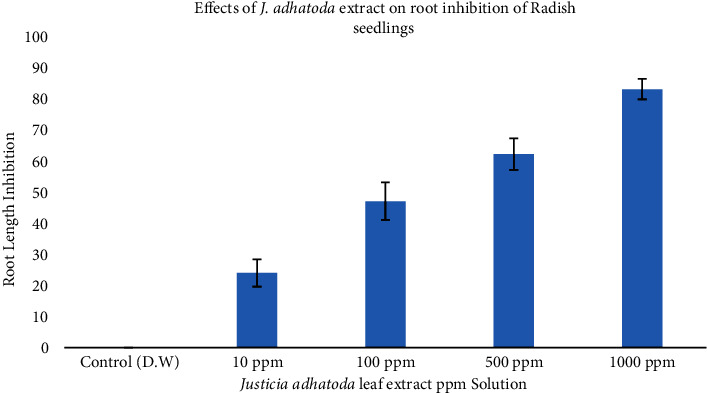
Effects of *J. adhatoda* extracts on root inhibition of Radish seedlings.

The effect of five concentrations (0, 10, 100, 500, and 1000 ppm) of *J. adhatoda* methanolic extracts on root length indicated that root inhibition increased by increasing concentration of extract (Tables 3 and 4, Figure 2). Root length of radish seedling decreased up to 83.11%. Means of root inhibition are 24.00%, 47.11%, 62.22%, and 83.11% in 10 ppm, 100 ppm, 500 ppm, and 1000 ppm extract, respectively (Figure 2).

Table 4 reveals the analysis of variance of effects of *J. adhatoda* extracts on root inhibition of radish seedlings. A significant difference between control and treated samples was observed.

Allelochemicals present in the plant extract might be responsible for inhibiting root growth in the plants being studied. These types of growth inhibition by allelopathic plants extract have also been reported by Islam et al. [48, 49].

They observed that the allelopathic activity of *Leucas aspera* is due to the presence of growth inhibitory substances. According to Devkota and Sharma, aqueous extracts of *J. adhatoda* and *Costus speciosus* plants slightly inhibited pea root and hypocotyl, at a 2% concentration. The inhibitory effects increased when the concentration was increased to 10%. Wheat root length decreased from 5.78 ± 1.33 cm in the control to 0.84 ± 1.96 cm at a 10% concentration of *J. adhatoda* leaves [44].

Shinwari et al. evaluated the allelopathic effects of 160 medicinal plants collected from Tanegashima, Japan. *Melia azedarach*, *Tylophora tanakae*, and *Cinchona* sp. have inhibitory potentials ranging from 80 to 100% against lettuce root development, while *Justicia procumbens* and 9 other plants show root inhibition between 60 and 79% [50]. These results are in agreement with the phytotoxic effect of *J. adhatoda* extract in our study. Gautam et al. investigated the allelopathic potential of six plants on radicle length of maize seeds. Among the six plants, *Parthenium hysterophorus* extract showed the maximum reduction in radicle length of maize seedlings and a mild concentration of *Lantana camara* extract induced the maximum reduction in plumule length [51]. Similar results were also found by Devi and Dutta that 10% concentration of *P. hysterophorus* foliar extract causes a high level of radicle growth inhibition of maize [52]. Our findings are in accordance with these previous studies which were planned to assess root length inhibition by allelopathic chemicals of different plants. Growth parameters (root and shoot length, seed germination) of other plants are inhibited by allelochemicals. Allelochemicals may change membrane permeability, inhibit nutrient uptake, inhibit cell division and elongation, change submicroscopic structure of cell, affect plant photosynthesis and respiration, change various enzyme functions, and also affect the synthesis of plant endogenous hormones and proteins [53].

### 4.3. Cytotoxicity Assessment of *Justicia adhatoda* Leaves Extract

**Figure 3:**
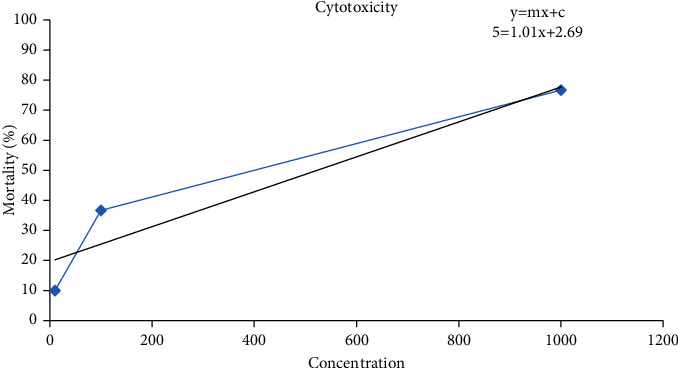
Cytotoxic analysis of *J. adhatoda* extracts.

The cytotoxic effect was studied by brine shrimp lethality assays. Concentrations of 10 ppm, 100 ppm, and 1000 ppm were tested to determine the mortality in percentage (%) of brine shrimp nauplii. The mortality was 10%, 36.67%, and 76.67%, respectively, for *J. adhatoda* at these concentrations (Table 5, Figure 3).

In investigation, the LC50 of *J. adhatoda* extract was 217 *µ*g/ml, which is considered to be cytotoxic.

Similar findings were also reported by Khan et al. [53]. They found that crude methanolic extract of *J. adhatoda* exhibited 13.33%, 3.33%, and 0% cytotoxicity at 1000, 100, and 10 g/ml, respectively [53]. Krishnaraju et al. revealed the results of screening of some medicinal plants for Brine shrimp cytotoxicity, in which *Aristolochia indica*, *Boswellia serrata*, *Garcinia cambogia*, *Ginkgo biloba*, and *Semecarpus anacardium* were shown to be considerably cytotoxic [54]. It was observed by Meskat and Hussain that the LC50 values of ethyl acetate, chloroform, and n-hexane-soluble fraction were found to be 1.402, 2.130, and 1.129 *µ*g/ml, respectively [55]. In a same way, Patel and Zaveri observed that the methanolic extract of *Justicia gendarussa* root and leaf showed a notable cytotoxic effect on brine shrimps. Active fraction of 100 *μ*g/ml dose level elicited 100% hatching inhibition and showed an LD50 value of 25.44 *μ*g/ml in the toxicity assay, which might indicate cytotoxic activity. The LD50 of the isolated compound from the toluene fraction was 8.13 g/ml [56]. Sadek investigated the impact of crude methanolic extracts of *Adhatoda vasica* leaves on the feeding of *Spodoptera littoralis* larvae. Feeding on fresh leaves caused 100% mortality of larvae after 26 days of insubstantial growth. The extract showed strong toxic and antifeedant activity against the larvae [57].

The cytotoxicity of gandarusa (*Justicia gendarussa* Burm.f.) was studied by Widodo et al. by Brine Shrimp Lethality Test (BSLT). They reported LC_50_ of 96% water extract and ethanol extract of *J. gendarussa* leaves to be 18.02 *μ*g/ml and 713.34 *μ*g/ml, respectively [58]. In mice, the hydro-alcoholic extract of *Justicia vahlii* has been found to be nontoxic up to 4000 mg/kg, and brine shrimp lethality assay showed no mortality [59]. We have observed significant cytotoxicity of 76.66% of *J. adhatoda* extract against brine shrimp at 1000 ppm concentration.

### 4.4. Proximates Analysis of *Justicia adhatoda* Leaves

Proximate analysis revealed that leaves of *J. adhatoda* have 9.4% moisture, 90.6% dry matter, 19.25% crude protein, 4.5% crude fat, 8.0% crude fiber, and 11.5% total ash (Table 6).

Similar to our findings, Baniya reported that the moisture content of leaves is 10.2% [60]. Our findings digress from those of Jayapriya and Shoba, according to whom the moisture content of *J. adhatoda* leaves was 18.20% [61]. Gulfraz et al. reported that *Adhatoda vasica* leaves had a 15.3% moisture content while roots had 24.6% [62]. According to Kumar et al., the moisture content of *J. adhatoda* leaves was 15.3 ± 0.5% [63]. Our findings revealed that plant leaf powder contains 90.6% dry matter, while according to Gulfraz et al., *J. adhatoda* have 50.4% and 66.4% dry matter in leaves and roots, respectively [62], which deviate from our findings. The dry matter content of *J. secunda* was also reported by Arogbodo to be 87.20 ± 0.25% [64].

The percentage of crude proteins in dried leaves of *J. adhatoda* is 19.25%. It is in conformity with Rasheed et al. [65]. They reported that *J. adhatoda* leaves had 21.33% protein content, which is almost near to our findings [65]. Gulfraz et al. reported that protein content was 8.5% in roots and 6.5% in leaves of *J. adhatoda* [62]. According to Kumar et al., the protein content of *J. adhatoda* leaves was 6.5 ± 0.3% [63]. The crude protein content of *J. secunda* was reported by Arogbodo to be 18.09 ± 0.18% [64]. Ajuru et al. showed that the leaves of *J. secunda* contain (22.33 + 0.02%) protein content, which was greater than the present findings of *J. adhatoda* protein content, and the leaves of *J. carnea* had a protein content of 17.53 + 0.02% [66]. This is slightly lower than our findings.

In our findings, crude fat percentage in dried leaves of *J. adhatoda* is 4.5%. Gulfraz et al. reported that fat content was 2.5% in roots and 1.6% in leaves of *J. adhatoda* [62]. According to Kumar et al., the crude fat of *J. adhatoda* leaves was 1.6 ± 0.3%. Our findings showed deviation from these findings [63]. Arogbodo reported that crude fat of *J. secunda* was 8.10 ± 0.39% [64].

Crude fiber percentage in dried leaves of *J. adhatoda* is 8.0. According to Kumar et al., the ash content of *J. adhatoda* leaves was 6.4 ± 0.45%, which is in harmony to our findings [63]. Arogbodo reported that crude fiber of *J. secunda* was 0.60 ± 0.14% [64]. Ajuru et al. reported that fiber content values of *J. carnea* and *J. secunda* were 42.53 + 0.00 and 42.21 + 0.02%, respectively, which were much greater than the present findings [66]. The percentage of total ash in dried leaves of *J. adhatoda* is 11.5%. Baniya reported similar findings, indicating that the total ash value was 13.3% [60]. Palshikar and Pandiyan reported that the ash content was 9.5% in *J. adhatoda* leaves of rainy season [67]. According to Gupta et al., physiochemical studies of *J. adhatoda* revealed that the plant contains 20% total ash, 82% acid-insoluble ash, and 4.5% water-soluble ash [68]. Jayapriya and Shoba narrated that the total ash content, water-soluble ash, and acid-insoluble ash of *J. adhatoda* leaves were not more than 21.40%, 4.85%, and 0.92%, respectively [61]. The ash content in *J. secunda* and *J. carnea* was reported by Ajuru et al., which was 15.62 + 0.03 and 15.01 + 0.01%, respectively [66]. Reddy et al. reported that the total ash content of *J. adhatoda* flowers was 6.89% [69].

### 4.5. Qualitative Analysis of Phytochemicals

**Figure 4:**
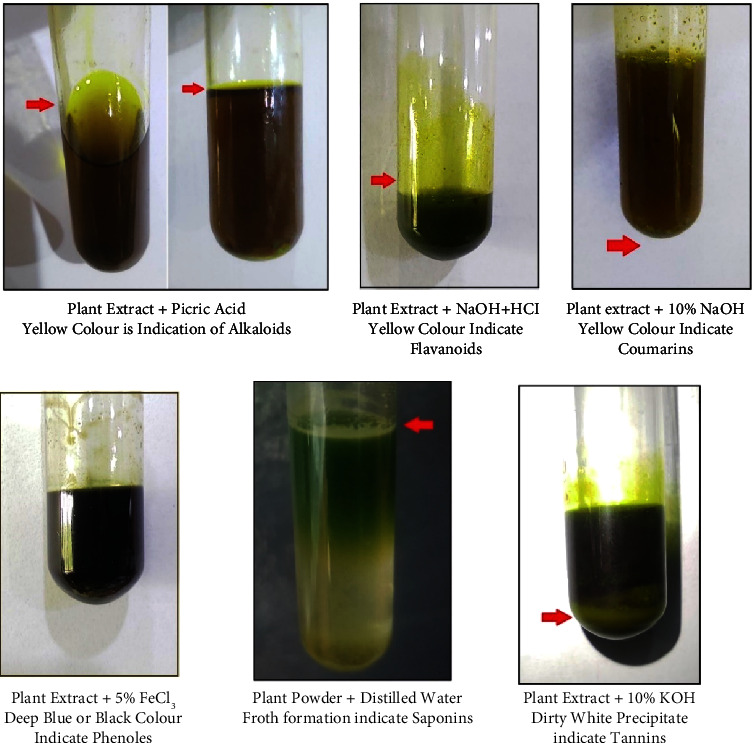
Results of different phytochemical test of *J. adhatoda* leaf extracts.

Plant extracts were scrutinized for the determination of different phytochemicals. Different phytochemical tests were performed to detect the presence of various phytochemicals. The results of phytochemical analysis of *J. adhatoda* leaves extract are shown in Table 7, which indicates the presence of various phytochemicals, such as alkaloids, coumarins, flavonoids, phenols, saponins, and tannins (Figure 4).

The phytochemical assessment was carried out on the crude methanolic extracts of *J. adhatoda* leaves. Our findings showed the presence of alkaloids, coumarins, flavonoids, phenols, saponins, and tannins. Similarly, Baniya indicated the presence of alkaloids, carbohydrates, cardiac glycoside, flavonoids, reducing sugar groups, steroids, saponins, tannins, and vitamin C in *J. adhatoda* leaves [60].

According to a group of Indian researchers, major chemical elements of *Adhatoda vasica* leaves include pyrroloquinazoline alkaloids, adhatonine, vasicine, vasicinone, vasicol, vascinolone, and vasicinol. Vasicine has been shown to have bronchodilatory, uterine, and respiratory stimulant properties. Vasicinone was also shown to have bronchodilatory and antianaphylactic action and weak cardiac stimulant [70].

The alkaloids have antioxidant, antidiabetic, antimicrobial, anti-inflammatory, antiallergic, abortifacient, uterine stimulant, bronchodilatory, and electrophoretic effects [71–73]. Vinothapooshan and Sundar reported that various leaf extracts of the plant *Adhatoda vasica* contain biologically active phytochemicals such as alkaloids, essential oil, flavonoids, quinazoline, tannins, and vasicinone, which are accountable for the significant hepatoprotective activity [74]. The flowers also contain a very good amount of phenolic compounds and flavonoid contents [75].

According to Chaudhary et al., phytochemical analysis of *Adhatoda vasica* leaves in methanolic extract, ethyl acetate, and aqueous extract exhibits the presence of saponins, carbohydrates, flavonoids, tannins, and alkaloids [76]. Similarly, according to Sarker et al., the ethanolic, petroleum ether, and water extracts of *Adhatoda* leaves are enriched with alkaloids, phenols, tannins, and reducing sugars. Main constituents like vasicine and others have been found to possess mild antibacterial activity against some microbes [77]. Vinothapooshan and Sundar suggested, according to their experimental data, that bioactive phytochemicals such as alkaloids, essential oil, flavonoids, quinazoline, tannins, and vasicinone present in the several extracts of the *Adhatoda vasica* plant may be responsible for the potential hepatoprotective activity [74].

Francis et al. demonstrated in experimental animals that saponins present in *Justicia* plants have antiviral, antifungal, antiprotozoal, and cytostatic activities on various types of cancer cells, cell-mediated immune system stimulation, lower serum cholesterol, and antibody production enhancement [78]. Tannins have strong antibacterial, antifungal, and antiviral properties as well as biological activity relating to their potential for protein precipitation and astringency, which have led its use as an antidiarrheal, wound healer, and antiseptic [79]. On the other hand, several coumarins have shown biological actions such as anti-inflammatory, anticoagulant, antioxidant, and analgesic activities [80]. The presence of these compounds assumes excellent pharmacological potential for *Justicia* species.

## 5. Conclusion

Proximate analysis revealed that leaves of *Justicia adhatoda* have 9.4% moisture, 90.6% dry matter, 19.25% crude protein, 4.5% crude fat, 8.0% crude fiber, and 11.5% total ash. The presence of various phytochemicals such as alkaloids, coumarins, flavonoids, phenols, saponins, and tannins was confirmed by employing different methods, which in turn confirmed the presence of these phytochemicals. Based on our findings, it is apparently clear that methanolic extracts of *J. adhatoda* leaves showed significant cytotoxic effects. *Justicia adhatoda* shows potential for use in antibacterial and antifungal medicine. In the present study, the *J. adhatoda* foliar extract shows significant inhibitory effects against radish seed germination and root growth. Further research is required to detect and isolate phytotoxins from this plant, which could potentially replace synthetic herbicides.

## Figures and Tables

**Table 1:** Tukey test results of the effect of *J. adhatoda* extract on germination of radish seeds.

Treatment	*N*	Mean (%)	Standard deviation	Grouping
0	3	93.667	1.528	A
10	3	85.33	2.52	AB
100	3	81.00	4.36	BC
500	3	75.67	4.51	C
1000	3	62.33	3.06	D

**Table 2:** ANOVA on effect of *Justicia adhatoda* extract on germination of radish seeds.

Source	DF	Adj SS	Adj MS	*F*-value	*P* value
Treatment	4	1638.9	409.73	35.73	0.001
Error	10	114.7	11.47		
Total	14	1753.6			

**Table 3:** Tukey test results of effects of *J. adhatoda* extracts on root inhibition of Radish seedlings.

Treatment (ppm)	*N*	Mean (%)	Standard deviation	Grouping
1000	3	83.11	3.29	A
500	3	62.22	5.09	B
100	3	47.11	6.01	C
10	3	24.00	4.37	D
0	3	0.000000	0.000000	E

**Table 4:** Analysis of Variance of effects of *J. adhatoda* extracts on root inhibition of Radish seedlings.

Source	DF	Adj SS	Adj MS	*F*-value	*P* value
Treatment	4	12614.5	3153.62	171.36	0.001
Error	10	184.0	18.40		
Total	14	12798.5			

**Table 5:** Cytotoxicity of *J. adhatoda* extracts on Brine shrimp nauplii.

Concentration	No. of nauplii taken	No. of nauplii dead	Total survivors	Mortality (%)	LC50
10	30	3	27	10	217
100	30	11	19	36.66666667	
1000	30	23	7	76.66666667	

**Table 6:** Proximate analysis of *Justicia adhatoda*.

Content	% Age (%)
Moisture	9.4
Dry matter	90.6
Crude protein	19.25
Crude fat	4.5
Crude fiber	8.0
Total ash	11.5

**Table 7:** Phytochemical analysis of *J. adhatoda*.

Name of compound	Result (presence/absence)
Alkaloid	Present
Coumarins	Present
Flavonoids	Present
Phenols	Present
Saponins	Present
Tannins	Present

## Data Availability

The data will be made available upon request to the corresponding author.
